# Supplementary material for: Deadly and venomous Lonomia caterpillars are more than the two usual suspects
Source: PLoS Negl Trop Dis. 2023 Feb 23;17(2):e0011063. doi: 10.1371/journal.pntd.0011063 (PMC9949635; doi:10.1371/journal.pntd.0011063)
Supplement: S1 Table — Abbreviations in the column headers are as follow: BIN: Barcode Index Number; Tree#: numbers refer to the position of each species in the neighbour joining tree of main text Fig 2; N: number of samples; N_bc: Number of DNA barcodes; Typ_bc: Type specimens sequenced (HT = holotype, LT = lectotype, PT = paratype); Ncountries: number of countries where the species is present; Countries: list of countries where the species was sampled (B = Bolivia, Be = Belize, Br = Brazil, C = Colombia, CR = Costa-Rica, E = Ecuador, FG = French Guiana, G = Guyana, Gt = Guatemala, H = Honduras, M = Mexico, N = Nicaragua, P = Panama, Pa = Paraguay, Pe = Peru, S = El Salvador, Su = Suriname, TT = Trinidad and Tobago, V = Venezuela); Dmax:: Maximum intraspecific distance (%); DminNN: Minimum distance to nearest neighbor; and NN: Identity of the nearest neighbor species. Species names in colored bold characters refer to species of confirmed (red) or suspected (orange) medical importance. Names in blue refer to provisionally named undescribed species. Gray rows highlight species with multiple BINs, and lines left of the species names connect those species pairs that share the same BIN. (PDF) [file pntd.0011063.s001.pdf]

Species Group	Species	BIN (as of JUL.15.2022)	Tree#	Authorship	N	N_bc	Typ_bc	Ncountries	Countries	Dmax	DminNN	NN	Notes
achelous	*Lonomia achelous*	ADG0261	**57**	(Cramer, 1777)	15	12		5	Br, C, FG, G, Su	0.8	1.2	*L. madrediosiana*	
	Lonomia annamiekae	AAY4841	**26**	Brechlin & Meister, 2013	2	2	HT	1	Pe	1.8	5.2	*L. viksinyaevi*	
	Lonomia belizonensis	ACF1945<br>AAB0791	**36**	Brechlin *et al.*, 2011	25	6<br>17	 HT	1	Br<br>FG	2	4.3	*L. canescens*	
	Lonomia beneluzi	AAK6621	**22**	Lemaire, 2002	10	5	-	1	FG	0	6.7	*L. venezuelensis*	
	Lonomia camox	AAE0724	**24**	Lemaire, 1972	37	27	HT	2	FG, V	4.2	3.3	*L. canescens*	
	Lonomia canescens	AAA8506	**25**	Brechlin & Meister, 2011	16	12	HT	3	B, E, Pe	0.8	3.3	*L. camox*	
	Lonomia casanarensis	AAB4839	**60**	Brechlin, 2017	27	25	HT	1	C	0.3	2.7	*L. diabolus*	
	Lonomia cayennensis	AAB0796	**42**	Brechlin & Meister, 2019	10	10	HT	2	C, FG	0.6	3.7	*L. CGR01*	
	Lonomia CGCM01	AAB4841	**49**	-	7	7	-	1	Br	1.2	3.7	*L. sinjaevorum*	
	Lonomia CGCM02	ABA7785	**43**	-	2	2	-	1	Br	0	3.4	*L. orientoandensis*	
	Lonomia CGR01	ACY4181	**44**	-	1	1	-	1	C	-	3.7	*L. cayennensis*	Caterpillar
	Lonomia CGR02	ADJ2216	**48**	-	1	1	-	1	Br	-	5.2	*L. sinjaevorum*	Caterpillar
	Lonomia descimoni	ACR7071<br>AAB0795	**37**	Lemaire, 1972	39	16<br>9	-<br>-	3	C, E<br>E, Pe	2.3	2.1	*L. rubrescens*	
	Lonomia diabolus	AAB4840	**59**	Draudt, 1929	27	23	-	3	FG, V, TT	1	2.7	*L. casanarensis*	stat. nov.
	Lonomia francescae	AAF5434<br>AAF5435	**51**	L. Racheli, 2005	7	2<br>4	-<br>-	1	E	3.9	2.3	*L. sinjaevorum*	
	Lonomia frankae	AAF5492	**27**	Meister *et al.*, 2005	16	3	PT	1	Pe	0.2	3.9	*L. pseudobliqua*	
	Lonomia madrediosiana	AAB4835	**58**	Brechlin & Meister, 2011	36	19	HT	2	E, Pe	1.5	1.2	*L. achelous*	
	Lonomia manabiana	AED0101	**32**	Brechlin *et al*., 2013	9	2	HT	1	E	0.6	1.8	*L. nigra*	includes *L. oroiana*, syn. nov. (HT with DNA barcode)
	Lonomia maranhensis	AAB4045	**56**	Brechlin *et al.*, 2011	10	10	HT	1	Br	0.8	3.5	*L. madrediosiana*	
	Lonomia moniqueae	AAP0951	**41**	Brechlin & Meister, 2019	9	6	HT	1	V	0.4	4.8	*L. diabolus*	
	Lonomia nigra	AEC7052	**33**	Brechlin *et al*., 2013	8	3	HT	1	E	0.3	1.8	*L. manabiana*	
	Lonomia orientoandensis	AAB4836	**45**	Brechlin & Meister, 2011	20	20	HT	3	C, E, Pe	2.6	3.4	*L. CGCM02*	
	Lonomia orientocordillera	AAB4838	**50**	Brechlin *et al*., 2013	22	17	HT	3	C, E, Pe	2.7	3	*L. sinjaevorum*	
	Lonomia panguana	ACL6324	**53**	Brechlin, 2017	3	3	HT	1	Pe	2	2.4	*L. riojensis*	
	Lonomia parubrescens	ABZ8294<br>ACE7193<br>ACF3136<br>ACK9075	**39**	Brechlin & Meister, 2011	22	3<br>9<br>3<br>2	HT	2<br>1<br>1<br>1	B, Pe<br>Pe<br>Pe<br>Pe	2.7	1.3	*L. rubrescens*	
	Lonomia pastazana	AAF5930	**47**	Brechlin, 2017	4	4	HT	1	E	0.6	0.5	*L. siriae*	
	Lonomia pseudobliqua	AAB0793	**28**	Lemaire, [1973]	7	4	-	1	Pe	0.2	1.6	*L. viksinyaevi*	includes *L. vikcuscensis*, syn. nov. (HT with DNA barcode)
	Lonomia puntarenasiana	AAA7086	**34**	Brechlin & Meister, 2011	35	31	HT	2	C, CR, N, P	2.6	1.8	*L. manabiana*	
	Lonomia rengifoi	ACS2525<br>ACS2526	**23**	Brechlin & Käch, 2017	4	3<br>1	HT	2<br>1	C, E<br>E	2.4	4.4	*L. canescens*	
	Lonomia riojensis	AAY4842	**52**	Brechlin & Meister, 2013	2	1	HT	1	Pe	-	2.3	*L. silkae*	
	Lonomia rubrescens	ACE5375	**40**	Brechlin & Meister, 2011	25	20	PT	1	Pe	0.5	1.3	*L. parubrescens*	
	Lonomia rubroguyana	AAB0790<br>ACV3238	**38**	Brechlin & Meister, 2019	32	26<br>6	HT	1<br>1	FG<br>FG	1.8	1.8	*L. parubrescens*	
	Lonomia rufescens	AAA7087	**31**	Lemaire, 1972	22	9	PT	2	C, E	0.8	4.8	*L. manabiana*	
	Lonomia serranoi	AAJ2304	**30**	Lemaire, 2002	22	13	HT	5	Be, Gt, H, M, S	2.8	5.4	*L. maranhensis*	includes *L. yucatensis*, syn. nov. (HT with DNA barcode)
	Lonomia silkae	ABZ8347	**54**	Brechlin & Meister, 2013	5	3	HT	1	Pe	0.5	1.7	*L. sinjaevorum*	
	Lonomia sinjaevorum	AAB0794<br>ACF0924	**55**	Brechlin & Meister, 2011	26	16<br>6	HT<br>PT	1<br>1	B<br>Pe	3	1.7	*L. silkae*	
	Lonomia siriae	AAF5930	**46**	Brechlin & Meister, 2013	22	14	HT	2	B, Pe	1.4	0.5	*L. pastazana*	
	Lonomia vanschaycki	AAB0792	**35**	Brechlin *et al*., 2013	37	17	HT	3	C, E, Pe	0.6	4.6	*L. canescens*	
	Lonomia viksinyaevi	AAB0793	**29**	Brechlin & Meister, 2011	5	5	HT	1	B	2.2	1.6	*L. pseudobliqua*	
electra	*Lonomia columbiana*	AAC9591<br>ABY3226<br>ACG1953<br>ADG2380	**6**	Lemaire, 1972	92	11<br>10<br>4<br>22	HT	2<br>1<br>1<br>2	C, E<br>CR<br>E<br>C, P	3.9	2.4	*L. laalbania*	
	Lonomia concordia	AAA5428	**7**	Druce, 1886	98	59	LT	2	CR, P	2.6	4.3	*L. quintanarooensis*	includes ssp. *conricana* (HT with DNA barcode)
	Lonomia electra	ABY6452<br>ABY6721	**14**	Druce, 1886	47	3<br>10	 LT	1<br>1	M<br>Gt	2.8	1.5	*L. luteomexicana*	
	Lonomia electra DHJ02	AAF5436	**8**	-	9	9	-	1	CR	0.3	2.9	*L. quintanarooensis*	
	Lonomia laalbania	ACO9490	**4**	Brechlin, 2017	2	2	HT	1	C	0	2.4	*L. columbiana*	
	Lonomia luteomexicana	ABY4571<br>ABY7782<br>ACE5178	**13**	Brechlin & Meister, 2011	7	2<br>3<br>2	 <br>HT	1	M	2	1.5	*L. paralectra*	
	Lonomia mexilectra	AAA6679<br>ABY8855	**10**	Brechlin & Meister, 2011	4<br>5	4<br>4	HT	1	M	0.8<br>1.8	2.6	*L. sinalectra*	 This BIN recognized as ssp. *mexpueblensis* (HT with DNA barcode)
	Lonomia minca	ACT2303	**1**	Brechlin, 2017	7	7	HT	1	C	0.2	4.8	*L. venezuelensis*	
	Lonomia paralectra	AAA6675<br>ADL2612	**12**	Brechlin & Meister, 2011	17	16<br>1	HT	1<br>1	Gt<br>M	2.3	1.5	*L. luteomexicana*	
	Lonomia peggyae	AAC9590<br>ACE5050<br>ACF7094	**5**	Brechlin *et al*., 2013	39	6<br>14<br>1	 HT	1	E	2.9	2.4	*L. columbiana*	
	Lonomia quintanarooensis	AAA6676	**9**	Brechlin & Meister, 2011	33	23	HT	5	Be, Gt, H, M, N	2.1	2.9	*L. electraDHJ02*	includes ssp. *quinchiapasiana* (HT with DNA barcode)
	Lonomia santarosensis	AAA7085	**3**	Brechlin & Meister, 2013	27	23	HT	3	CR, H, N	2.2	3.5	*L. columbiana*	
	Lonomia sinalectra	AAA6678<br>AAA6680<br>ABA7784<br>ABY6181<br>ABY6182<br>ACE3733<br>ACF3079<br>ACZ2305	**11**	Brechlin & van Schayck, 2015	3<br>1<br>3<br>2<br>2<br>2<br>2<br>1	16	HT	1	M	4.3	2.6	*L. mexilectra*	
	Lonomia venezuelensis	AAC1812	**2**	Lemaire, 1972	34	29	-	2	C, V	0.9	3.5	*L. laalbania*	
obliqua	*Lonomia antoniae*	ABA3772	**15**	Brechlin & Meister, 2015	31	11	HT	1	Br	1.4	5.2	*L. obliqua*	
	Lonomia CGCM03	ABA7783	**16**	-	3	3	-	1	Br	0.2	1.3	*L. leopoldina*	
	Lonomia leopoldina	AAC1813<br>ACV8217	**17**	Brechlin & Meister, 2011	9	4<br>1	HT	1	Br	2.6	1.3	*L. CGCM03*	
	Lonomia obliqua	AAB4042	**20**	Walker, 1855	49	33	-	3	B, Br, Pa	1.8	1.8	*L. parobliqua*	
	Lonomia parobliqua	AAB4043<br>ACE6785	**21**	Brechlin *et al.*, 2011	37	22<br>12	HT	3<br>4	A, Br, GF<br>A, B, Br, Pa	2.2	1.8	*L. obliqua*	
	Lonomia RRBR01	AAB4044	**18**	-	13	9	-	1	Br	1	6.7	*L. obliqua*	
	Lonomia rufobahiana	ABA3773	**19**	Brechlin & Meister, 2013	4	3	HT	1	Br	0.3	3.9	*L. obliqua*	
