# Supplementary material for: Deadly and venomous Lonomia caterpillars are more than the two usual suspects
Source: PLoS Negl Trop Dis. 2023 Feb 23;17(2):e0011063. doi: 10.1371/journal.pntd.0011063 (PMC9949635; doi:10.1371/journal.pntd.0011063)
Supplement: S2 Fig — This panel shows the distribution of each one of the seven (7) Lonomia species belonging to the Obliqua group. The inset maps show the countries in South and/or Central America where the occurrence records have been recorded and a square is present in the inset maps in case a more precise information of the location within the country is needed. The map in the bottom right corner shows the distribution of the species richness of this group at a resolution of 400km2 grid cell. Country border shape file available at: https://gadm.org/data.html. (PDF) [file pntd.0011063.s004.pdf]

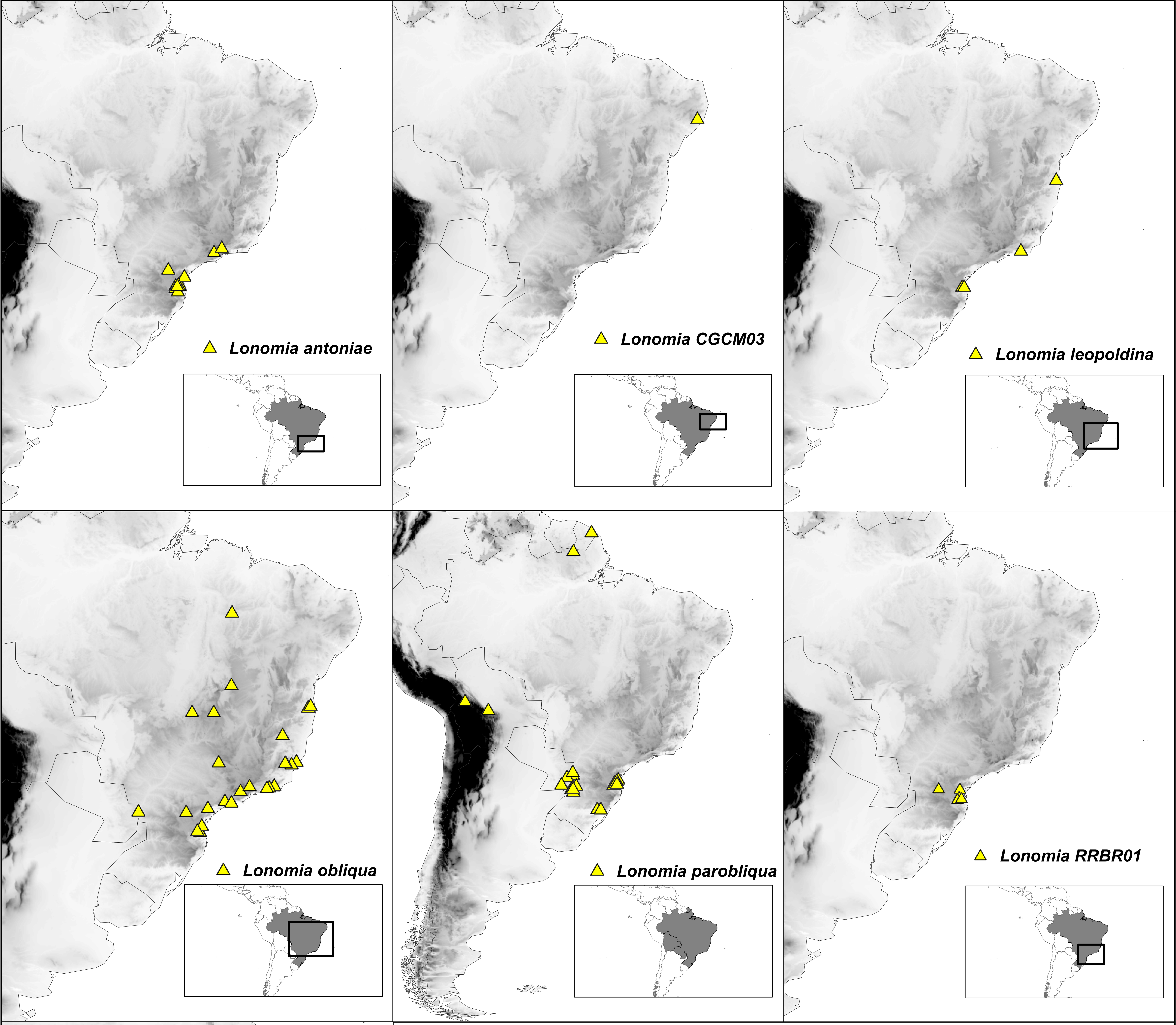

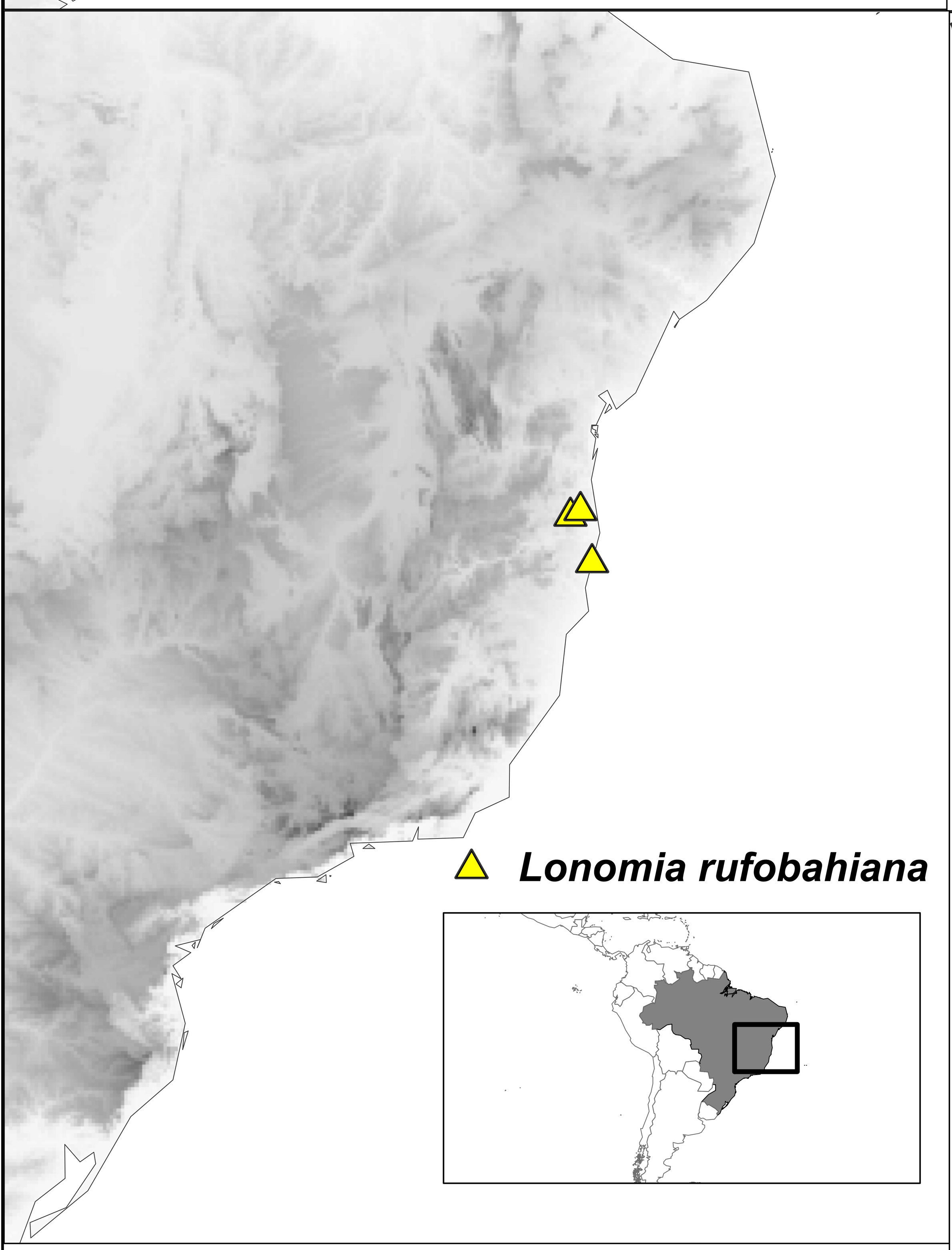

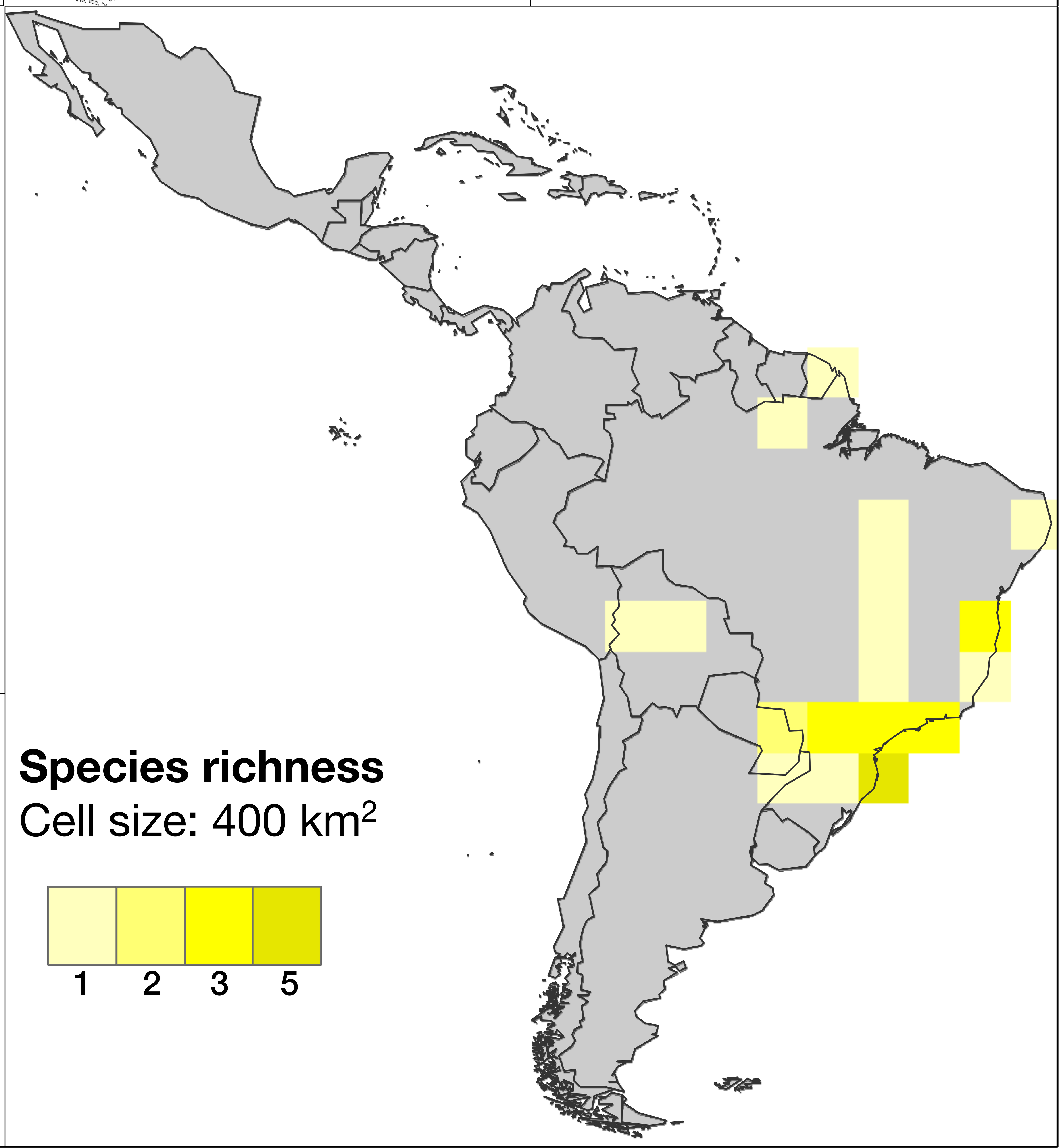

Supplementary Fig. S2. Obliqua Group
This panel shows the distribution of each one of the seven (7) *Lonomia* species belonging to the Obliqua group. The inset maps show the countries in South and/or Central America where the occurrence records have been recorded and a square is present in the inset maps in case a more precise information of the location within the country is needed. The map in the bottom right corner shows the distribution of the species richness of this group at a resolution of 400km^2 grid cell.
