# Supplementary material for: Deadly and venomous Lonomia caterpillars are more than the two usual suspects
Source: PLoS Negl Trop Dis. 2023 Feb 23;17(2):e0011063. doi: 10.1371/journal.pntd.0011063 (PMC9949635; doi:10.1371/journal.pntd.0011063)
Supplement: S3 Fig — This panel shows the distribution of 13 species of Lonomia belonging to the electra-group as defined in the present study. The inset maps show the countries where occurrence records are known. The bottom right map shows species richness of the group at a resolution of 400km2 grid cell. Country border shape file available at: https://gadm.org/data.html. (PDF) [file pntd.0011063.s005.pdf]

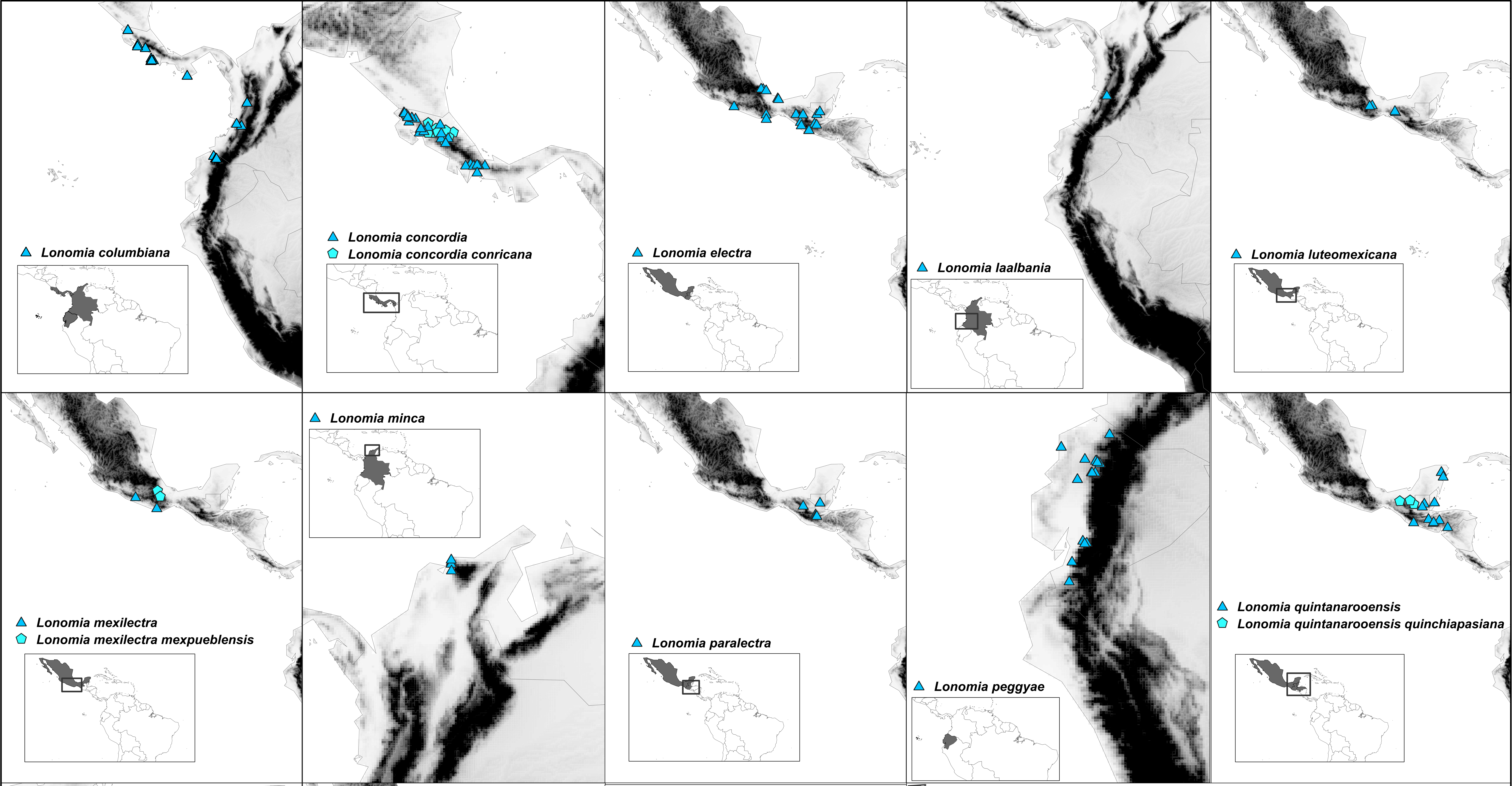

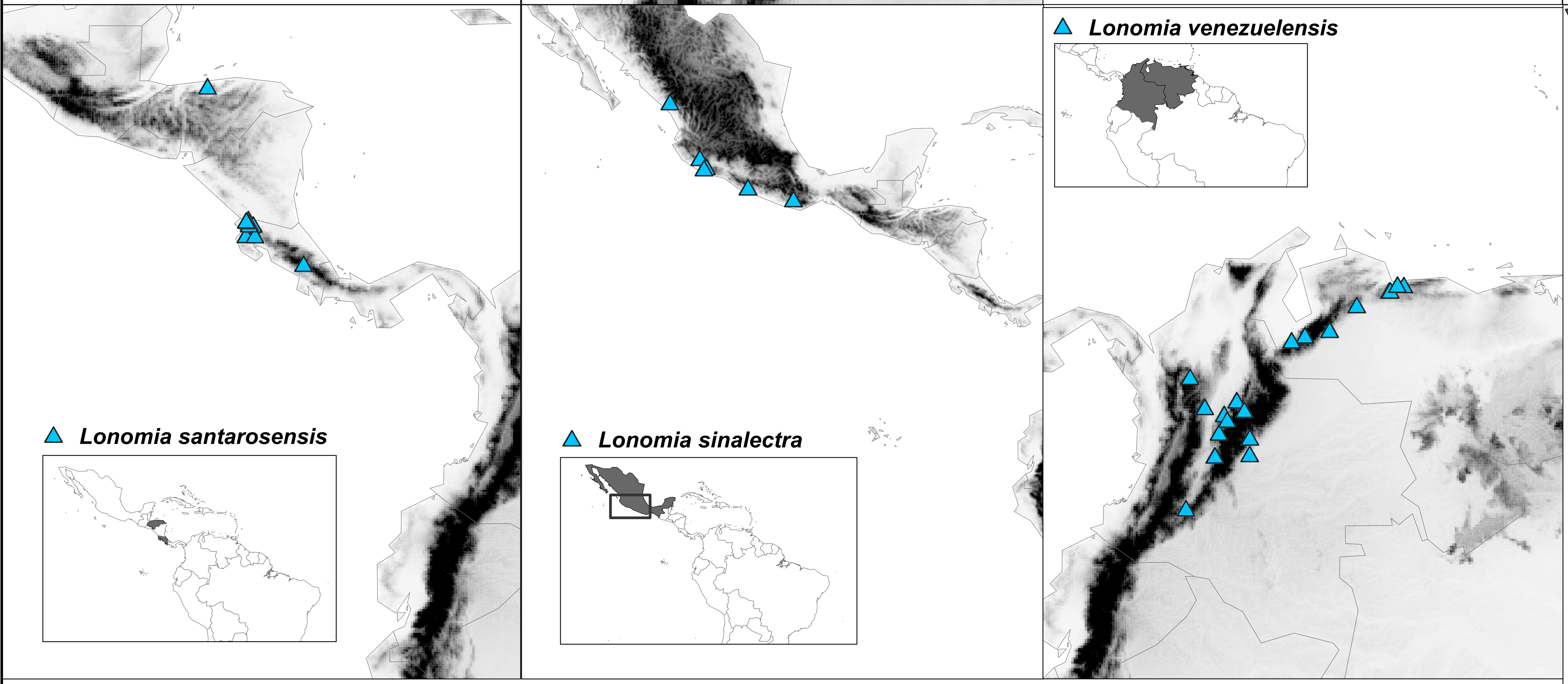

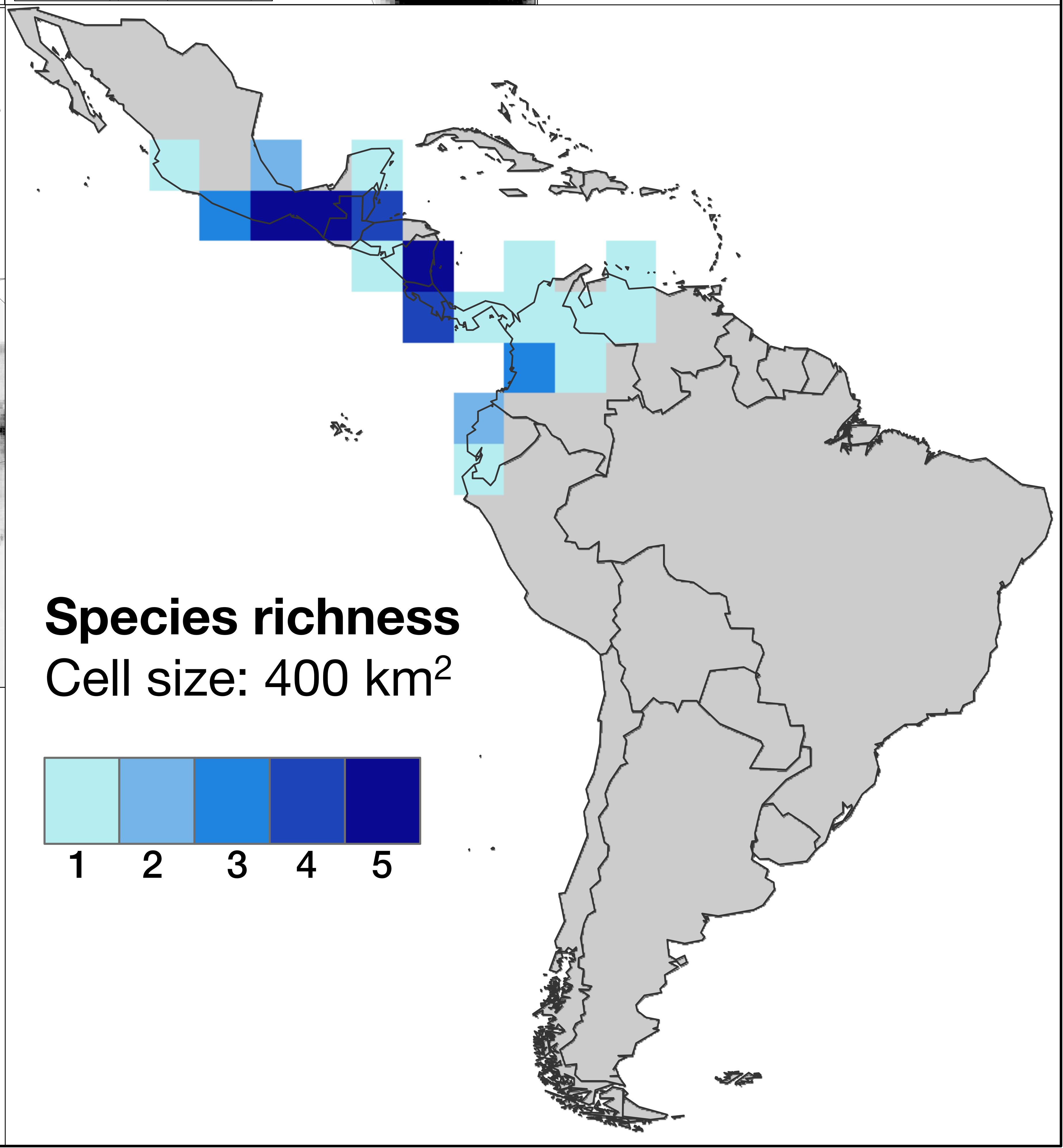

Supplementary Fig. S3. Electra Group

This panel shows the distribution of 13 species of *Lonomia* belonging to the electra-group as defined in the present study. The inset maps show the countries where occurrence records are known. The bottom right map shows species richness of the group at a resolution of 400km^2 grid cell.
